# Implementing a Picture Prompt and Proximity Intervention in a Classroom with an Adult Learner: A Case Study

**DOI:** 10.1007/s40617-024-00924-2

**Published:** 2024-03-06

**Authors:** Todd Haydon, Christina Carnahan

**Affiliations:** University of Cincinnati, CECH, Teachers College, 2600 Clifton Avenue, Cincinnati, OH ML0022 USA

**Keywords:** Classroom management, Inclusion, On-task behavior, Picture prompt, Teacher proximity

## Abstract

The current case study investigated the use of a picture prompt and teacher proximity with an adult learner with significant disabilities during a postsecondary classroom management class and generalization setting. Results indicated that the learner had a higher percentage of intervals of on-task behavior during intervention than baseline. Teacher and learner satisfaction ratings suggested that the intervention was an acceptable strategy. A discussion of study limitations, implications, and future research directions are included.

• A simple low tech instructional strategy can improve student on-task behavior.

• Teachers can use an antecedent strategy to improve student behavior.

• Strategies used in K–12 environments may work in higher education settings.

• Successful applied behavior analysis strategies help include students in group participation.

The Higher Education Opportunity Act of 2008 (HEOA) was authorized to enable institutions of higher education (IHEs) to create or expand high-quality inclusive programs for students with intellectual disabilities (ID) and developmental disabilities (DD). Over the past decade, inclusive postsecondary opportunities have become available to students with intellectual and developmental disabilities. These programs have demonstrated strong outcomes in employment both during college and upon graduation (Grigal et al., 2018). Experiences that focus on academic enrichment, socialization, independent living skills, integrated work experiences, and career skills lead to employment opportunities (National Core Indicators, 2019).

Although inclusion is important, the interventions used must have ecological validity. That is, be effective, feasible, acceptable, cost-effective, have generality, and take place in the natural environment (Fahmie et al., 2023). It is surprising that there is a lack of research exploring specific behavioral interventions that address staying on task, acquiring job skills, and learning academic content at the postsecondary level.

One strategy that offers a solution is the use of prompts. However, results concerning the type of prompt are mixed. Teacher verbal prompts have been effective with preschool students with disabilities and in combination with teacher proximity with elementary school and middle students in general education classrooms (Faul et al., 2012). However, Steed and Lutzker (1997) demonstrated that an adult with a disability learned to complete vocational tasks using picture prompts compared to vocal prompts. Ingvarsson and Hollobaugh (2011) demonstrated that picture prompts resulted in fewer trials to mastery criterion in learning to respond correctly to questions than vocal prompts with three boys (4 years old) with autism. To fill this gap in the literature, the present study addressed the call for additional research.

## Research Questions

The following research questions of the study were proposed:

- What is the effect of using a picture prompt card and proximity on the on-task behavior of a student identified with a significant disability during an undergraduate classroom management class?
- What is the effect of a picture prompt card and proximity in a generalized setting?

## Method

### Participants and Setting

#### Student

Stu (name is a pseudonym), age 21, entered the university program with a diagnosis of significant disability based on a high school evaluation team report (ETR). Selecting and managing course work was part of the educational programming of the PSE program. Therefore, Stu advocated for and requested to enroll in the behavioral management class and gave assent to participate in the study. The class was Stu’s first college class and he needed support with accessing and learning course content. He would sleep during class, look out the window, and occasionally laugh out loud, most likely to gain peer attention.

According to the *Kaufman Test of Education Achievement* (Kaufman & Kaufman, 2004), form A, Stu read at a 6th–8^th^ grade level, his reading comprehension standard score was 59, and listening comprehension was 76 (average range: 85–115). Stu independently used a power chair to arrive at his classes and communicated through an augmentative and alternative communication (AAC) device. Stu typed his responses on the AAC, including the tests and social validation scores.

### Teacher, Job Coach, and Settings

The classroom teacher was an associate professor, Caucasian male with 10 years of teaching experience at the higher education level. The study took place in an applied behavior analysis (ABA) class required for a junior special education cohort. There were approximately 34 students in the class during the study. The study took place during whole group teacher-led instruction at the same time each morning. An entire class session typically lasted 130 min; however, the 30-min teacher proximity and picture prompt intervention was used only during the beginning of class time. The study was conducted over a period of 12 weeks with sessions occurring once a week. The job coach was a senior undergraduate special education student. The generalization setting was a local drugstore where Stu worked once a week for 2 hr. Data collection in this setting was also 30 min in duration at the beginning of the shift.

### Materials

The authors used a picture prompt card that was 3.5 x 6.5 in. The teacher selected lesson objectives from chapters in the book *Managing Classroom Behavior Using Positive Behavior Supports* (Scott et al., 2011). To measure improvement in learning the instructor administered a pre- and posttest. Questions were taken from the book *Applied Behavior Analysis for Teachers* (9^th^ ed.; Alberto & Troutman, 2012). The same questions were used for the pre- and posttest.

Pre- and posttest scores were measured as the percentage of correct responses as well as the number of items correct on 18-item multiple choice quizzes. The same test was administered on the first and last days of class. During the pretest and posttest, students were not allowed to use the textbook. According to Stu’s accommodation plan, a university professor independently administered the tests in another setting on campus. As part of his established academic programming, Stu used a “look back” procedure (a type of error correction strategy used in the college’s postsecondary program) from the Qualitative Reading Inventory-5 (QRI-5) reading assessment to retrieve answers from the textbook on 9 out of 18 questions during the posttest.

### Data Collection and Training

Three advanced undergraduate students trained in ABA served as observers and were trained by the first author. To control for observer drift, the teacher met with the three observers on a biweekly basis for retraining. The third observer was the job coach. To ensure accuracy of pre- and posttest scores a faculty member who did not participate in the study independently scored the pre- and posttest. All observations lasted a total of 30 min and sessions occurred after the first 5 min of class. A 1-min momentary time sampling recording system was used for on-task behavior. During this time the teacher’s use of proximity and picture prompts was recorded for treatment adherence.

### Dependent Measures

#### On-Task Behavior

On-task behavior included using the AAC device (for Stu) and for everyone, looking at and/or writing responses on a worksheet, head and body facing peers while discussing academic material, and tracking the teacher during teacher-led instruction (Faul et al., 2012). On-task behavior also included Stu’s answering questions on signal and following teacher directions related to the material being covered in class. In the generalization setting, on-task behavior was defined as engaging in the task (i.e., stocking shelves, picking up boxes), eyes on the job coach when given directions, and eyes on the task.

### Interobserver Agreement

Interobserver agreement was assessed only in the classroom setting for 100% of baseline and intervention sessions by having a second observer simultaneously collect data. On-task behavior was calculated using an interval agreement formula, the number of intervals of agreement, divided by the total number of intervals and multiplied by 100%. The unit of measurement was percentage of 1-min intervals. Mean interobserver agreement for on-task behavior across the two study conditions was 91.1% and 94.6% (range: 90.6%–100%). Total count interobserver agreement for correct answers (smaller sum divided by larger sum) was used for pre- and posttest scores. Interobserver agreement was 100%.

### Experimental Design and Procedure

An alternating treatments design with a baseline phase throughout the study was used to assess the effectiveness of the intervention. The two conditions were alternated each class meeting. Phase changes were determined by a minimum of three data points during baseline.

### Baseline

During this condition, the teacher lectured on ABA content, used questions to assess student understanding, and showed videos of behavioral concepts. The students volunteered responses by raising their hands. The teacher remained at the front of the room near his desk and did not use teacher proximity or the picture prompt for the entire session. Baseline for the generalization setting consisted of the job coach supervising Stu at the drugstore and observing him from approximately 7 ft while he stocked shelves. Teacher proximity with the picture prompt card was not used. After three data points were collected the teacher and job coach implemented proximity with the picture prompt strategy.

### Training Session with Picture Prompt

The teacher had previously attended a 1-day training on the Good Behavior Game including a 15-min presentation on the use of picture prompts. After baseline and before the first intervention data point, the teacher read a script to Stu and the class that covered the procedures of the picture prompt: (1) introduced OK/Not OK by referring to the lanyard card; (2) explained to the student and class that you will use the picture prompt to send messages; (3) provided examples of messages, “When you see me touch ‘OK,’ it means you’re on task and should keep it up! If you see me touch ‘Not OK,’ you are off-task and think about what needs fixing; (4) the teacher modeled some behaviors that may receive “OK” or “Not OK”; (5) the teacher used OK/Not OK cues without verbal interruptions that distracted the rest of the students in the classroom. Stu used his AAC device indicating a “Yes” that he understood each procedure. The training took approximately 5–10 min to complete.

### Picture Prompt and Proximity Intervention

During this condition the teacher followed the procedures during baseline except he wore a Motivaider device that vibrated every 5 min. After the cue of the Motivaider he walked within an arm’s length of Stu pointed towards “OK” portion of the card when Stu was on task or pointed to “Not OK” when Stu was off task. The procedure lasted for approximately 5 s. To avoid stigmatization the teacher also used the picture prompt with the entire class. The classroom was divided into six quadrants. After cueing Stu, the teacher stood in each quadrant for approximately 5 s and pointed to the card in view of the group of students. The teacher did not systematically use proximity for the other individual students in the classroom. In the generalization setting the job coach followed the same procedures as the classroom teacher.

### Treatment Adherence

A procedural checklist was developed to measure the accuracy with which the teacher used the picture prompt and proximity intervention. Independent observers checked each step as either present or absent before each intervention session. Checks for the intervention were scored during 100% of sessions and consisted of the five steps listed above. The observers also marked whether the teacher pointed to “OK” or “Not OK.” Treatment adherence was calculated by dividing the number of steps present by the total number of steps and then multiplying by 100%. Treatment integrity data indicated that the teacher implemented all procedural steps of the teacher proximity with picture prompt with 100% adherence on all occasions.

### Social Validity

At the completion of the study, the teacher and Stu completed a 5-question social validity survey regarding the acceptability and usefulness of the teacher proximity with picture prompt strategy. The survey consisted of 4-point Likert scale responses indicating 1 (not at all), 2 (somewhat), 3 (fairly), 4 (very). The questions consisted of the ease of implementation, perceived intervention effectiveness, and likelihood of future intervention use.

## Results

**Fig. 1:**
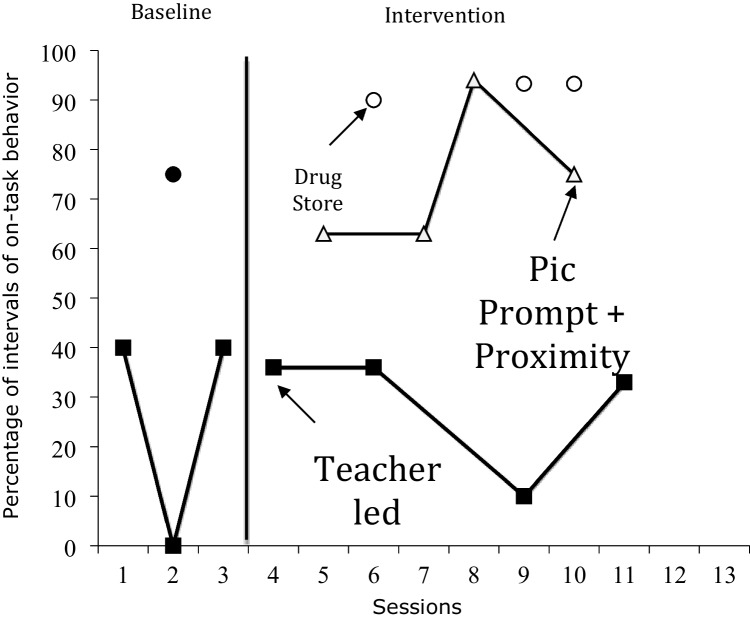
Percentage of Intervals of On-Task for Stu in the Classroom, and Generalization (Drugstore) Settings across Baseline (Closed Symbols) and Intervention (Picture Prompt + Proximity; Open Symbols) Phases

The percentage of intervals of on-task behavior for Stu during class and generalization setting across all phases of the study is shown in Fig. 1. During baseline (closed squares) Stu’s mean percentage of intervals of on task behavior was 27.9 % (range: 0.0%–40.0%.) and 75.0% during the probe generalization setting (closed circles). During intervention (open triangles) the mean percentage of intervals for Stu was 73.8% (range: 63.0%–94.0%), and during the generalization setting (open circles) was 92.2% (range: 90.0%–93.3%). Stu displayed the lowest levels of on-task behavior during the baseline condition. In contrast, he showed a fairly higher level of on-task behavior during intervention and a slightly higher level during the generalization setting. Further visual analyses revealed that for Stu four out of four (100.0%) intervention data points exceeded the highest baseline data point.

Additional data were collected on test outcomes. The mean percentage of pretest scores was 0.0% for Stu and the class. Mean posttest scores for the class was 16 out of 18, 88.8% (range: 83.3%–94.4%) and posttest score for Stu was 17 out 18 (94.4%).

Regarding preference of conditions, Stu indicated that he liked the picture prompt and proximity over baseline. Regarding whether he liked the nonverbal feedback or no feedback at all, Stu indicated that he liked the nonverbal interaction and feedback of being on task or off-task. He indicated (4.0) that the thumbs up on the picture prompt helped him do better in class. Regarding his perception of being on task he gave (4.0) for the intervention. The teacher indicated that teacher proximity was very easy to implement (4.0), was very effective (4.0), and was very likely to use the intervention in the future (4.0).

## Discussion

The positive results of the study may be examined from the viewpoint of ecological validity (Fahmie et al., 2023). The study was conducted in a typical higher education classroom setting and work setting (i.e., drug store). The instructor used typical teacher led instruction, a common ABA textbook, and materials. The selection of on-task behavior was meaningful for Stu. Furthermore, his percentage of intervals of on-task behavior (92.2%) was highest during the generalization setting. Thus, the strategy was also effective outside the confines of the experiment. Ecological validity was enhanced in that Stu selected the course and agreed with the prompting strategy and behavioral goal. The intervention offers a strategy for college professors to implement for including students with significant disabilities. Finally, anecdotally, the amount of time Stu slept in class was greatly decreased.

Although the results of the posttest cannot be contributed to the intervention, the results provide some indication that the intervention helped support Stu in accessing course content and that he learned behavioral concepts at the college level.

### Limitations and Future Research

Despite the positive findings of this study, a few limitations should be considered. Treatment adherence and IOA data were not conducted in the generalization setting; future researchers could collect these data. To build on this preliminary study, future studies could (1) include students with higher rates of challenging behavior; (2) evaluate the effects of the intervention upon the whole class; (3) investigate implementing the intervention with more participants with various disabilities; (4) examine learning as an actual dependent variable, such as daily quiz scores; (5) include a maintenance phase; and (6) compare proximity alone with proximity and a picture prompt.

In summary, the picture prompt card and proximity strategy allowed Stu to be engaged and learn college level ABA content. The positive effects of the intervention carried over to the generalization setting, which had an impact on acquiring job skills that are necessary to be successful in the work environment.

## Data Availability

The authors confirm that all data generated or analyzed during this study are included in this published article. The datasets generated during the current study are available from the corresponding author on reasonable request.
